# Provision of ankle foot orthoses for children with cerebral palsy in Norway

**DOI:** 10.1177/20556683241276804

**Published:** 2024-09-27

**Authors:** Tobias Goihl, David F. Rusaw, Karin Roeleveld, Siri Merete Brændvik

**Affiliations:** 1 Department of Neuromedicine and Movement Science, Faculty of Medicine and Health, Norwegian University of Science and Technology, 8018 NTNU, Trondheim, Norway; 2 Trøndelag Orthopaedic Workshop, TOV, Trondheim, Norway; 3 Department of Rehabilitation, 4161 Jönköping University, Jönköping, Sweden; 4 Clinical services, St.Olavs University Hospital, Trondheim, Norway

**Keywords:** Clinical practice, recommendations, guidelines, clinical decision-making, orthotic prescription, ankle-foot orthosis (AFO), cerebral palsy

## Abstract

**Introduction:**

Practice of ankle-foot orthoses (AFO) provision for ambulatory children with cerebral palsy is underreported and the literature is not consistent on choice of AFO-design. This study describes clinical practice of AFO provision for children with cerebral palsy and evaluates how clinical practice aligns with existing recommendations.

**Methods:**

An online, cross-sectional survey was conducted, inviting all Norwegian orthotists working with children with cerebral palsy. Orthotic practice was investigated using a self-reported survey design.

**Results:**

From all eligible orthotists, 54% responded, revealing that AFO provision involves patients, physicians, and physiotherapists at different stages. Patient preference directly influenced the ultimate AFO-design. Shank vertical angle was evaluated by 79%. For children with crouch gait and those with short gastrocnemius, a majority preferred a combination of rigid and articulated/flexible AFO-designs. Instrumented gait analysis was conducted by 51% at AFO delivery stage.

**Conclusions:**

The findings show that AFO provision in Norway is collaborative, involving clinical team members and consideration of patient preferences. A discrepancy between clinical practice and existing recommendations for children with crouch gait and those with short gastrocnemius is observed.

## Introduction

Ankle-foot orthoses (AFO) play a significant role in clinical management of children with cerebral palsy (CP) where they are frequently used to improve or maintain gait function^1^ and optimize quality and energy efficiency of gait by controlling ankle-foot alignment.^2^ The World Health Organisation (WHO) states the importance of evidence-based practice and has established standards for orthotic service delivery^3^ that describe patient assessment, fabrication and fitting, user training and product delivery, and follow-up as key-stages in AFO provision, where goal setting and outcome evaluation are included as part of these standards. The International Organization for Standardization (ISO) provides the definition of AFO as “orthoses that encompass the ankle joint and the whole or part of the foot”^4^ but does not provide specific definitions for the numerous AFO-designs that are available to improve gait functions. Terminology for AFO-designs varies considerably^5^ and for the purpose of this study the AFO-designs have been categorised into two types based on available ankle motion: rigid AFOs, which prevent all ankle movement, and articulated/flexible AFOs, that have a mechanical articulation or are constructed from a flexible material, allowing varying degrees of ankle movement.^6^ The latter group contains AFOs with different properties which may restrict or assist plantar- and/or dorsiflexion.

One approach for choosing the most suitable AFO-design is based on designs proposed for each of the following six gait patterns: drop foot, true equinus, genu recurvatum, jump gait, apparent equinus and crouch.^7^ Several proposals agree on the use of rigid AFOs for crouch.^8–11^ However, whilst rigid AFOs have been proposed for true equinus,^8,10^ genu recurvatum,^8,10^ jump gait,^8,10^ and apparent equinus,^8–10^ there exists little consensus in the literature, as articulated AFOs have also been proposed for true equinus,^8,9^ genu recurvatum,^8,9^ jump gait^8,9^ and apparent equinus.^8^ Moreover, a systematic^12^ and scoping^13^ literature review have described benefits on gait kinematics of both, rigid and articulated AFOs for children with crouch and equinus gait without establishing specific AFO-design recommendations.^12,13^ However, findings in both literature reviews and primary studies have been called into question.^13,14^ Reflecting this uncertainty, Firouzeh et al.^15^ and Owen^11^ have reported that improvements in gait kinematics cannot be linked to specific AFO-designs due to a lack of evidence. The ambivalence in the literature on the benefits of specific AFO-designs for specific gait patterns might limit the use of this approach as clinicians may have difficulty in choosing amongst conflicting evidence.

Another approach for choice of AFO-design is based on the length of gastrocnemius, where short gastrocnemius describes a condition where muscle function is impaired due to contracture, spasticity, or stiffness.^16,17^ In this specific condition, rigid AFOs are recommended with shoe modifications to optimize their alignment.^18^ This recommendation was initially formulated in 2008, at a consensus conference of the International Society for Prosthetics and Orthotics (ISPO)^18^ and has been further developed into a design algorithm^19^ and prescription principles.^10^ Based on this work, rigid AFO footwear combinations (AFO-FC) have been shown to improve gait kinematics, function, and mobility in children with short gastrocnemius and are thought to preserve triplanar stability of the foot.^20–23^ These two approaches are not mutually exclusive and can be combined.

The AFO-design process requires both biomechanical and clinical expertise and is determined by treatment goals, which in turn are influenced by gait pattern and length of gastrocnemius as well as patient preferences. Gait pattern should be evaluated during initial assessment, to aid choice of AFO-design, and at the stage of AFO delivery, to determine whether the AFO facilitates the attainment of these goals. For reliable results, use of video or video vector analysis has been recommended.^24^ Consideration of patient preferences and realities of the families are also essential for the AFO-design process^11,15^ and these change during childhood.^11,25^

Thus, although recommendations exist, no widely adopted, evidence-based practice guidelines have been developed for choice of AFO-designs in the treatment of children with CP. The use of AFOs is therefore mainly based on clinical, practice-based expertise and patient preferences.^11^ In Norway, orthotists are part of a multidisciplinary team and play a central role in the design process and it is not uncommon for them to simply receive a prescription for an “AFO” from a physician, without detailing the design. However, it is not known how AFO provision standards are implemented into clinical practice or how clinical practice aligns with available recommendations. The first aim of this study is therefore to describe AFO provision practice among Norwegian orthotists. The focus is on assessment, fabrication and fitting, and delivery. The second aim is to evaluate how current orthotic practice aligns with the recommendations for crouch and short gastrocnemius and discuss possible discrepancies.

## Methods

To address the study aims, we developed a cross-sectional survey focusing on AFO provision (patient assessment and AFO fabrication, fitting, and delivery). To assess whether children’s preferences and requirements change over the course of childhood in a way which influences AFO-design, we asked for age specific AFO properties. The topics of patient training and follow-up are recognized as important but are excluded from the survey as they lie beyond the scope of this study. In line with previous work, the questionnaire was developed by the authors who are clinical experts and researchers in the field.^24^ Six orthotists with a minimum of 7 years of clinical experience from five clinics in Norway completed the first draft of the questionnaire to evaluate ease of understanding, terminology, and use of language. Individual interviews were conducted and based on the feedback, three orthotists assessed a revised version. Following another round of interviews, the final questionnaire was developed, and an English translation is provided in Table 1. Ethical approval from the research ethics board for the survey was not required because it falls outside the Norwegian health research act^26^ and participation was anonymous.

**Table 1.** Questions used in the survey.

Topic	Questions		Format	Options
Background information on respondents				
	1	For how many years have your worked as orthotist?	S-MC	0-1, 1-3,3-5,5-10,>10
	2	For how many years have you worked with children with CP?	S-MC	0-1, 1-3,3-5,5-10,>10
	3	How many children with CP do you usually see in your clinic per week?	3-LS	<1, 1-3, >3 per week
	4	Do you feel confident in the process of fitting AFOs to children with CP?	3-LS	Little confident/confident/very confident

AFO provision: Assessment, fabrication and fitting, delivery				
Assessment for AFO design	5	How often are AFO-goals specified by the referring physician?	4-LS	N-S-U-A
	6	Do you usually specify AFO-goals for a child that is to receive an AFO if this has not been done already?	4-LS	N-S-U-A
	7	How frequently do you discuss AFO-goals with the child/caregivers/referring physician and physiotherapist?	MQ	N-S-U-A
	8	How frequently does the physician in your clinic specify that an AFO should also prevent foot deformities?	4-LS	N-S-U-A
	9	How do you usually analyse a patients gait pattern for the choice of an appropriate AFO? (Visual/Video/Video-Vector/Report from 3DGA)	MQ	N-S-U-A
	10	If you could choose, what would you do to analyse a patients gait pattern in your clinic?	OEQ	
	11	Which AFO properties do you think are important for a 5-year-old attending kindergarten?	OEQ	
	12	Which AFO properties do you think are important for a 10-year-old attending school?	OEQ	
	13	Which AFO properties do you think are important for a youth?	OEQ	

	Indicate which AFO-design you find appropriate for each of the gait patterns drop foot, equinus, genu-recurvatum, jump-knee, apparent equinus, crouch			
	14	Flexible, no articulation, prefabricated (such as ToeOff©)	MQ	
	15	Flexible, no articulation, individually made (e.g. Springer©)	MQ	
	16	Articulated, open dorsiflexion, plantarflexion-stop	MQ	
	17	Spring loaded articulation (e.g. NeuroSwing©, TrippleAction©)	MQ	
	18	Rigid AFO, no ankle movement	MQ	
	19	*If you have other comments for choice of AFO design for different gait patterns*, *you can write them here*	OEQ	
AFO practice for children with short gastrochnemius	20	In cases where the clinical examination shows 5 degrees dorsiflexion with flexed knee and 5 degrees plantarflexion with extended knee: is this an important indication for choice of rigid AFO without ankle movement?	Yes/No	
	21	*If yes*, *how would you set the ankle angle in the AFO? (dorsiflexion/neutral/plantarflexion)*	S-MC	In dorsiflexion/neutral/plantarflexion
	22	*If familiar with tuning*: *When you choose not to fit rigid AFO-FC with shoe modifications for children with tight gastrocnemius*, *what are the reasons?*	M-MC	^a^
	23	*Other reasons for not fitting rigid AFO-FC*	OEQ	
AFO fabrication & fitting	24	With whom do you discuss the mechanical properties of the AFO: With the child/caregivers, referring physician, physiotherapist	MQ	N-S-U-A
	25	Do you evaluate the shank vertical angle for control of alignment when the child walks?	4-LS	N-S-U-A
	26	How do you evaluate shank vertical angle?	OEQ	
	27	Are you familiar with the method of “tuning” rigid AFO-footwear combinations (AFO-FC) that accommodate tight gastrocsoleus and achieve correct shank vertical angle by changing the sole of the shoe?	Yes/No	
	28	*If yes*: *Have you fitted rigid AFOs with the ankle set into plantarflexion that were “tuned” by modifying the sole of the shoe?*	Yes/No	
	39	*If yes*: *how often to you fit tuned AFO-FC with shoe modifications for children with tight gastrocnemius?*	4-LS	N-S-U-A
	30	*If yes*: *how do you find correct alignment when you tune AFO-FC with shoe modifications?*	M-MC	Visual/Video/Video-vector/Report from 3DGA
	31	Which other criteria do you use to make sure you get good AFO-alignment?	OEQ	
AFO delivery	32	Do you specifically evaluate whether AFO-goals have been met?	4-LS	N-S-U-A
	33	How do you assess whether an AFO works as intended?	M-MC	ask child/caretaker, visual observation; Video; video-vector; report from 3DGA; other
	34	*If “Other”*: *How do you evaluate that an AFO works as intended?*	OEQ	

*Italics* symbolizes dependent questions. Abbreviations: S-MC, single-answer multiple choice; M-MC, multiple-answer multiple choice; OEQ, open ended questions; 3-LS, three-point Likert scale; 4-LS, four-point Likert scale; N-S-U-A, Never-Sometimes-Usually-Always; 3DGA, three-dimensional gait analysis.

^a^Choose one or several: I prefer AFO with articulation in these cases as well/“tuning” works in theory, but in practice we cannot change all the patients’ shoes/Low acceptance for AFO-FC with patients/Fitting takes too much time/I don’t have the equipment/I lack expertise/Other.

The percentage of respondents giving a particular answer were calculated for (a) the background information of the respondents and (b) AFO provision including assessment for AFO-design, AFO fabrication and fitting, and AFO delivery. The questionnaire contained multiple choice questions, dichotomous questions, matrix questions and open-ended questions. For questions related to AFO-designs for specific gait patterns (questions 14-18), the percentage of respondents using a specific AFO-design for each gait pattern was calculated. The full Norwegian questionnaire is available (Supplemental).

The survey was accessible between February and June 2022 via NETTSKJEMA^©^ (University of Oslo, Norway^27^). The National Association of Prosthetic/Orthotic Clinics in Norway, Ortopeditekniske Virksomheters Landsforbund (OVL), provided an overview of all clinics in Norway that employ orthotists. All clinics reported the number of orthotists working with children with CP and distributed the questionnaire among them. In addition, we promoted the questionnaire through the National Association of Prosthetists and Orthotists, NITO Ortopedi. Both organizations sent out reminders after three weeks.

After closing the survey, data were exported into Microsoft Excel, where descriptive analysis was conducted. Two of the authors (TG and SMB) reviewed the open-ended questions. The responses provided consisted of limited words or short sentences and lacked the richness relevant for qualitative analysis.^28^ As such, they were coded into explicit categories with an inductive approach.^29^ The questionnaire refers to children and teenagers up the age of eighteen, for simplicity referred to as “children”. “Respondents” refers to the orthotists that participated in the survey.

## Respondents

Responses from all 16 Norwegian orthotic clinics showed that 80 orthotists work with paediatric orthotics, of which 43 (54%) participated in the survey. Most respondents had worked as orthotists for more than 10 years (77%, Table 2) and 65% had worked with children with CP for more than 10 years. 47% of respondents describe themselves as confident when fitting AFO to children with CP and 51% as very confident.

**Table 2.** Clinical experience of respondents and level of confidence (in %).

Clinical experience			Clinical experience with children with CP			Confidence when fitting AFO to children with CP		
In years	Number of responses (%)		In years	Number of responses (%)		I feel	Number of responses (%)	
<1	1	(2%)	<1	2	(5%)	A little confident	1	(2%)
1-3	4	(9%)	1-3	4	(9%)	Confident	20	(47%)
3-5	2	(5%)	3-5	2	(5%)	Very confident	22	(51%)
5-10	3	(7%)	5-10	7	(16%)			
>10	33	(77%)	>10	28	(65%)			
total	43	(100%)	total	43	(100%)	total	43	(100%)

## Results

**Figure 1.**
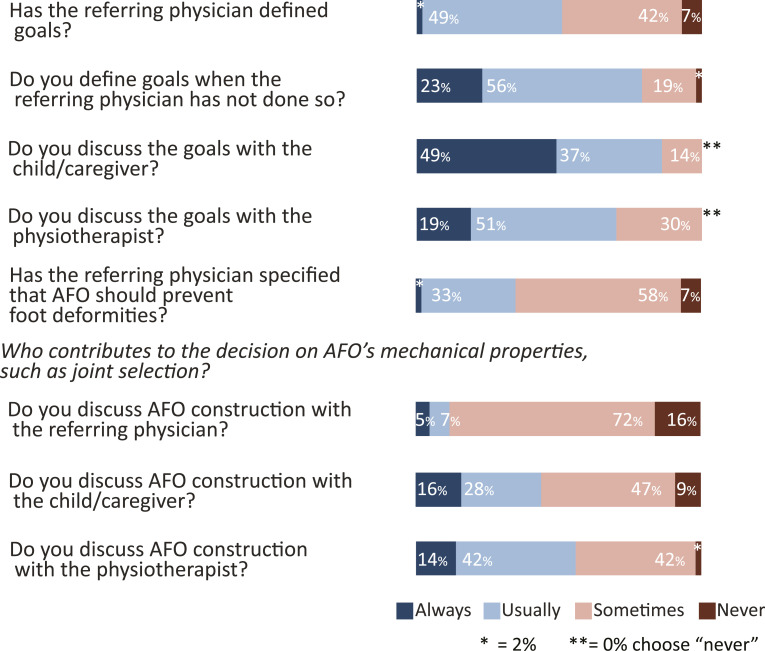
Goal settings and decision on AFO’s mechanical properties. Responses are given in % for “always”, “usually”, “sometimes” and “never”.

*Questions on assessment* for AFO-design focused on goal setting, gait analysis for AFO-design, patient preferences, and AFO-designs for different gait patterns. 49% of the respondents report that referring physicians usually define goals for AFO and 2% always. 56% usually set AFO goals if physicians have not done so and 23% always do so (Figure 1). Respondents discuss AFO goals with the child and/or the caregivers (37% usually, 49% always) and with the physiotherapist (51% usually, 19% always). About one third report that physicians also specify that AFOs should prevent foot deformities (33% usually, 2% always).

**Figure 2.**
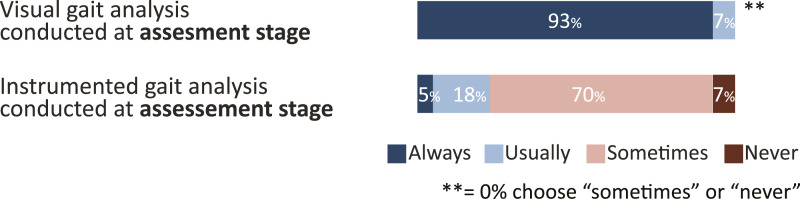
Method of gait analysis to aid choice of AFO-design at assessment stage. The options video, videovector and report from 3D gait analysis were summarized into “Instrumented gait analysis”. Responses are given in % for “always”, “usually”, “sometimes” and “never”.

During assessment stage, gait analysis is conducted visually (7% usually, 93% always) to aid choice of AFO-design while few routinely use instrumented gait analysis (18% usually, 5% always) (Figure 2). When asked, 65% of respondents describe that their preferred method of gait analysis is video or video vector and 35% do not provide an answer.

Patient preferences affect AFO design and are influenced by patient’s age. The replies consisted of common themes that were summarized for each age group: For five-year-old children, half of the respondents (51%) report that AFOs must not limit activity or transitions from playing on the ground to standing and walking. These respondents prefer articulated solutions. In addition, it had to be easy to use AFOs correctly for rotating kindergarten-staff who help the children with use of their AFO (reported by 42%). AFO should be slim, lightweight and have an appealing design (reported by 37%).

For 10-year-old children, nearly half of the respondents (47%) highlight the importance of children being able to use AFO independently. Particularly in school-time, where children in Norway change between indoor and outdoor shoes for longer breaks, “*it is important not to be always last in the school yard*” as one respondent writes. Appearance is important (reported by 37%), and AFO function (such as gait improvement, improved stability, and lower energy cost) has to be noticeable, as children reflect more on why they use AFOs (reported by 40%). For teenagers, half of the respondents (51%) specify that an AFO must have the biomechanical function that the child wants to have. Just as many report the importance of finding a design that the child finds acceptable. Respondents write that an AFO must “*function in daily life*” and it “*should work for the whole family*”.

Choices of rigid and/or articulated/flexible AFO-designs for each of six gait patterns are shown in Table 3. The most frequently chosen AFO-designs for Drop foot are prefabricated flexible AFO (84%) and articulated AFO with plantarflexion stop and free dorsiflexion (67%). For the other gait patterns, a combination of articulated/flexible and rigid AFO-designs is chosen. For Equinus and Crouch gait, more than 50% choose rigid AFO and at least one type of articulated AFO. With one exception, all respondents select rigid AFO for at least one gait pattern.

**Table 3.** AFO-designs for six different gait patterns.

AFO design	Varying degrees of ankle movement	Drop foot 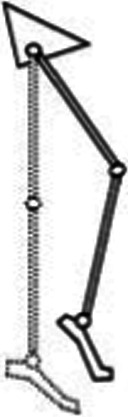	*Equinus 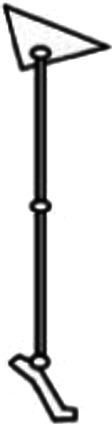	*Genu Recurvatum 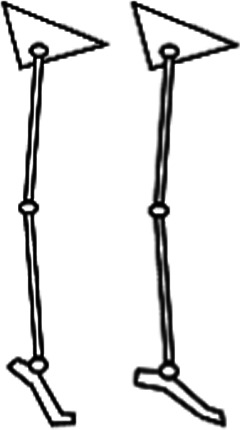	*Jump Gait 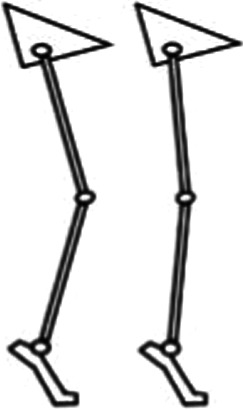	Apparent Equinus 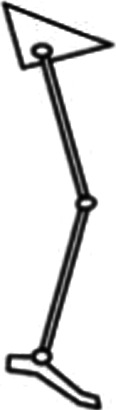	Crouch 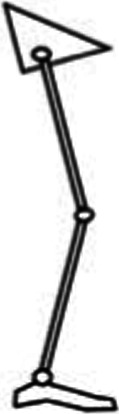	Not used for any pattern
**Flexible AFO**								
no ankle joint, prefabricated	yes	**84 %**	9 %	16 %	2 %	30 %	37 %	2 %
**Flexible AFO**								
no ankle joint, custom made	yes	30 %	28 %	30 %	23 %	35 %	40 %	26 %
**Articulated AFO**								
free dorsiflexion, plantarflexion stop	yes	**67 %**	**70 %**	**70 %**	33 %	12 %	2 %	0 %
**Articulated AFO**								
spring loaded joint	yes	21 %	56 %	**74 %**	53 %	51 %	60 %	9 %
**Rigid AFO**								
no ankle joint	no	2 %	53 %	35 %	37 %	44 %	**79 %**	2 %

Respondents were asked which AFO-designs they thought appropriate for each gait pattern. Each AFO-design was matched with one, several, or none of the six gait patterns. The numbers indicate the percentage of respondents choosing a certain AFO-type for a specific gait pattern. Choices made by more than 66% are highlighted. The one AFO-design that does not allow ankle movement is shaded. Illustration of gait pattern printed with permission (Papageorgiou, 2019).

* = for these gait patterns, the survey specified that the ankle was in equinus in stance or that reduced dorsiflexion limited tibia progression in stance.

**Figure 3.**
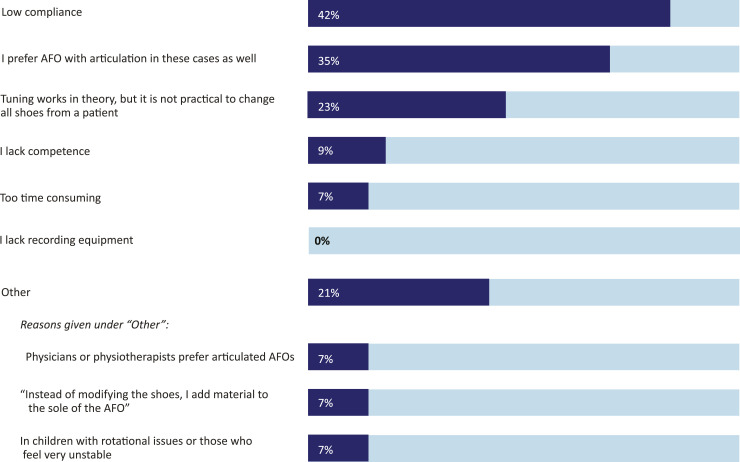
Reasons for not fitting solid AFO-footwear combination (AFO-FC) with shoe modifications for children with short gastrocnemius (in %). Respondents who chose “other” could specify their reason in a free text field. These answers are summarized in three topics.

*In the context of AFO practice for children with short gastrocnemius*, most of the respondents (60%) do not interpret this condition as an indication for rigid AFO and of those who do, the majority (13 out of 17) would set the ankle angle of the AFO into plantarflexion. Of all respondents, 93% state that they are familiar with the methodology of tuning rigid AFO footwear combinations (AFO-FC) with shoe modifications and most (72%) have fitted rigid AFOs with the ankle set into plantar flexion. Reasons for not fitting rigid AFO-FC with shoe modifications for children with short gastrocnemius (Figure 3) are: low compliance among children (42%), respondents’ preference for articulated AFO (35%) and practical issues (23%). Lack of recording equipment (0%), lack of competence (9%) or time constraints (7%) are not reported frequently. Further details are provided in free text answers, such as different opinions on the benefit of rigid AFO-FC among members on the clinical team (7%).

*For AFO fabrication and fitting*, details about the mechanical properties of AFOs are discussed with physiotherapists (28% usually, 16% always) and children (42% usually, 14% always) more than with referring physicians (7% usually, 5% always) (Figure 1). Shank vertical angle is evaluated to control AFO alignment during walking (51% usually, 28% always). Plum-line, goniometer, laser, or an app for bench- and static alignment is used by 21%.

**Figure 4.**
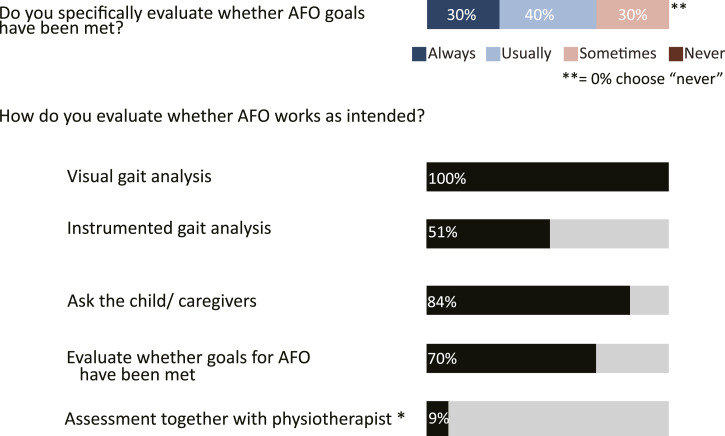
Goal evaluation and methods for evaluating whether AFO works as intended at delivery stage. For goal evaluation, respondent choose between “always, usually, sometimes, never”. For the remaining questions, one or several of the listed option could be chosen. The options video, video-vector and report from 3D gait analysis were summarized into “Instrumented gait analysis”. Responses given in %; * from open ended question.

*For AFO delivery*, 40% usually evaluate specifically whether AFO goals have been met and 30% always do so (Figure 4). To evaluate whether AFO works as intended (with regards to alignment and gait), all respondents analyse gait visually, 51% conduct instrumented gait analysis and 84% ask the children/parents. 9% specify that AFO evaluation is done together with a physiotherapist.

## Discussion

The aim of this survey was to document the practice of orthotists in the provision of AFO for ambulatory children with CP in Norway. The response rate (54%) was high compared to a previous study^24^ and contains the majority of the entire population of interest. The results are not intended as a prescription guide, but as a contribution to the ongoing discussion of how to implement evidence in clinical practice.

Goal setting for AFO is done in a collaborative process involving discussions with the children and their caregivers (86% usually or always do this) and physiotherapists (70% usually or always do this), which is in line with Kane and co-workers.^30^ The findings that only 51% of physicians usually or always communicate goals when prescribing AFO and only 12% usually or always discuss mechanical properties suggests the need for better communication within the clinical team about the intention and function of AFOs.

To capture aspects of patient preferences we explored age-related factors that have an influence on AFO-design. In younger children, respondents of our survey balance the advantages of rigid AFO-FC against the perception by the clinical team that articulated AFOs allow more activities. While this concern has been described before,^13,31,32^ it is not backed up by evidence. As AFO use peaks in 5-year-olds,^33^ when children are in kindergarten, more knowledge about AFO impact on activity and its effect on foot deformities are of great importance. In older children, respondents wrote that independent handling of AFOs becomes important. They comment that, with increasing age, children reflect more on their appearance and why they use AFOs. “*An AFO must function in daily life*” as one respondent writes. The respondents describe the importance of children understanding what the AFO is supposed to do and that the effect must be noticeable for their activities. While the importance of including children in the provision process has been described in general terms before,^11^ responses from the current survey highlight specific areas that may affect design choices.

The method of gait analysis was investigated twice, for the assessment stage (Figure 2) and for the delivery stage (Figure 4). During assessment, gait analysis is largely done visually to aid choice of AFO-design. While a majority (65%) report video or video-vector as their preferred method of gait analysis, it is not known why the 70% who use it “sometimes” do not use it more often. During AFO delivery, to evaluate whether AFOs work as intended, 51% report the use of instrumented gait analysis, which is higher than the 30% reported by Kane et al.^30^ While most report to evaluate shank vertical angle of the AFO during walking, it is unclear how this can be achieved adequately through visual gait analysis. Video-based analysis has been recommended for better reliability.^24^ Most respondents assess goal attainment and communicate with the child and caregivers, which has been reported as essential.^11^ However, no specific outcome measures were identified and information on actual performance in daily life, which should be part of an evaluation,^8,15^ is lacking.

To investigate whether there were preferences for specific AFO-designs based on gait pattern alone, we asked respondents to match AFO-types with different gait patterns. For the gait patterns Equinus and Crouch, more than 50% of respondents select a combination of articulated and rigid AFOs . While this finding shows a clear deviation from the recommendations of rigid AFO for crouch^8–10^ it is possible that respondents associate crouch with a spectrum of severities, ranging from those with very slight increase in knee- and dorsiflexion to those with severe flexion pattern, requiring different AFO-designs. While Bayon et al^8^ also list articulated AFO as an option for equinus, genu-recurvatum, jump-knee and apparent equinus, and Rodda et al.^9^ do so for true equinus and jump gait (in hemiplegia), they do not consider the impact of short gastrocnemius on the development of foot deformities which is emphasized by Owen^11^ and Wright.^10^ The variety of AFO-designs chosen for each gait pattern might show the width of individual presentations associated with each pattern.

The use of rigid AFO-FC to accommodate short gastrocnemius (as seen in equinus, recurvatum and jump gait) has been widely reported in recent years^6,11,18,22^ with a sound theoretical framework to support this approach.^10^ Contrary to these recommendations, 60% of respondents do not see short gastrocnemius as an indication for rigid AFO-FC. To accommodate short gastrocnemius, shoe modifications and heel wedges of up to 6,5 cm have been reported.^6^ Our respondents report compliance issues for such solutions. When respondents choose between rigid AFO-FC and articulated AFO-designs, it is unclear how much weight is given to possible long-term effects on foot deformities or whether some respondents work according to older research, which describes the concept of stretching a tight gastrocnemius through dorsiflexion whilst supporting the midfoot against collapse.^34^ In 2002 Morris et al.^35^ explained variations in the use of rigid AFOs by differences in professional preference and use of a biomechanical and a neurodevelopmental therapy-orientated programme. However, since the 2008 ISPO consensus,^18^ recommendations have been clear on the use of rigid AFOs for children with short gastrocnemius. In our study, only one-third of physicians are reported to specify that AFO goals should include the prevention of foot deformities, a finding that might reflect uncertainty of AFO effectiveness in this area. In this context, it is problematic that no literature review or clinical study has clearly documented benefits of orthotic management of foot deformities for children with CP.^12,18,36,37^ We found a preference for AFO-designs of respondents that allows more ankle movement than the recommendations for crouch and short gastrocnemius outline. The effect of this approach on alignment, foot stability and activity is not known. Additionally, patient preference for different AFO-designs is not well documented, although one pilot study found that functional benefits of AFO-FC outweighed cosmetic concerns.^23^ The possibility of maintaining ankle movement has become easier by the development of new articulation systems^38^ and use of functional electrical stimulation as an alternative to traditional AFO.^39^ However, new components and technologies should not be implemented at the expense of foot stability or optimal thigh- and shank alignment. We found that orthotists have a central role in individualizing patient care, which also emphasises their responsibility to communicate how to achieve good function, alignment, and protection against foot deformities. While the role of orthotists might be different in other countries, the general challenges in the decision-making process for the optimal AFO-design are likely to be similar. As long as individualization of AFO provision is primarily based on clinical expertise and patient preferences^11^, evaluation and documentation of outcomes remain critical.

Our study complements the broader perspectives of technology and interdisciplinary care^8^ and parents’ perspectives^15^ reported previously. It adds insights into provision practice of different AFO-designs which is not reported in population-based studies,^1,33^ highlights discrepancies to existing recommendations, and identifies specific areas of research that could help to align clinical practice with these recommendations. Some considerations are necessary when interpreting the results. The small size of the total orthotist population makes generalization challenging, despite a response rate of more than 50%.^40^ Nonetheless, the respondents’ uniform background (state authorized orthotists), similar work environment, and extensive work experience lend credibility to the responses.

The results from our survey indicate, that an effort should be made to align clinical practice with available evidence in the AFO-design, particularly for children with short gastrocnemius. This is supported by research which shows that clinical practice may not be optimal.^2^ However, the path from evidence to decision is not clear as most literature reviews on interventions for children with CP were considered unreliable and flawed.^14^ The Grading of Recommendations, Assessment, Development and Evaluation (GRADE^41^) system has been used to formulate strong recommendations based on low quality evidence because it considers benefits and harms, quality of evidence, values, acceptability, feasibility, equity, and cost. GRADE recommendations for AFOs for children with CP would provide a transparent method to address areas of concern and discrepancy between practice and evidence. The benefit of GRADE was recognized, and the Norwegian Registry for Cerebral Palsy, NOR-CP, is currently in the process of developing knowledge-based guideline for diagnosing and monitoring individuals with cerebral palsy which will describe important principles of orthotic management as well.^42^

## Conclusion

Responses from this survey show that AFO provision practice in Norway is multifaceted and collaborative. During assessment, gait is evaluated visually and treatment goals for AFO are formulated. Respondents reported different preferences for different ages which affect AFO-design. During fabrication and fitting, evaluation of shank vertical angle is conducted by the majority. During the delivery phase of provision, only half of respondents reported conducting instrumented gait analysis although this has been proposed as necessary to attain optimum function. For the spectrum of crouch gait, a majority use both rigid AFOs and designs that allow varying degrees of ankle motion. ISPO recommendations to accommodate short gastrocnemius in rigid AFOs were not adopted by most respondents. Thus, discrepancies between current clinical practice and existing recommendations are observed.

## Supplemental Material

Supplemental Material - Provision of ankle foot orthoses for children with cerebral palsy in Norway Supplemental Material for Provision of ankle foot orthoses for children with cerebral palsy in Norway by Tobias Goihl, David F. Rusaw, Karin Roeleveld and Siri Merete Brændvik in Journal of Rehabilitation and Assistive Technologies Engineering
