# Supplementary material for: Provision of ankle foot orthoses for children with cerebral palsy in Norway
Source: J Rehabil Assist Technol Eng. 2024 Sep 27;11:20556683241276804. doi: 10.1177/20556683241276804 (PMC11440621; doi:10.1177/20556683241276804)
Supplement: Supplemental Material - Provision of ankle foot orthoses for children with cerebral palsy in Norway [file sj-pdf-1-jrt-10.1177_20556683241276804.pdf]

Supplemental Digital Content: the complete questionnaire in Norwegian
The questions evaluated in this study are labelled with their corresponding number (Table 1). Some questions were not included in the current work because they fell outside the scope of the research question.

AFO til gående barn med CP- dagens praksis

Takk for at du tar deg tid til å delta på denne spørreundersøkelsen. Ditt bidrag er veldig viktig, og sammenstillingen av svarene skal deles med alle verksteder og ortopediingeniører.
Hensikten med dette arbeidet er å **kartlegge dagens praksis** for ortopediingeniører som lager og tilpasser ankelfotortoser (AFO) til gående barn (0-18 år) med cerebral parese.
Det finnes ingen fasit-svar. Vi ønsker bare å forstå hvordan du jobber!
Dersom du ikke jobber med denne pasientgruppa i dag, trenger du ikke svare på undersøkelsen.
Den vil ta omtrent 30 minutter å fullføre. Det er ikke mulig å mellomlagre. Hvis du må gå ut av skjemaet, må du begynne på nytt. Undersøkelsen er helt anonym; det er ikke mulig å spore hvem som har svart eller hvor svaret kommer fra.
Svarene fra undersøkelsen vil inngå i et PhD arbeid for undertegnede og planlegges brukt i en av artiklene som inngår i PhD arbeidet. Kopi av den aktuelle artikkelen vil bli tilsendt alle ortopediske verksteder i Norge, tentativt høsten 2022.
Hvis du har noen spørsmål, er det bare å ta kontakt!

Obligatoriske felter er merket med stjerne*

Bakgrunnsinformasjon

1 **Hvor mange år har du jobbet som autorisert ortopediingeniør?***

Turnus

0-1 år

1-3 år

3-5 år

5-10 år

i mer enn 10 år

2 **Hvor mange år har du jobbet med barn med CP?***
Vi ønsker å vite i hvor mange år du har jobbet med barn og unge som har cerebral parese.

0-1 år

1-3 år

3-5 år

5-10 år

i mer enn 10 år

3 **Hvor mange barn og unge med CP arbeider du med i en normal arbeidsuke?***

mindre enn 1 per uke 1-3 per uke

flere enn 3 per uke

mindre enn 1 per uke 1-3 per uke

4 Opplever du deg selv som trygg i arbeidsprosessen med å tilpasser AFOer til barn/unger med CP? *

**Jeg opplever meg lite trygg**	**ganske trygg**	**Jeg opplever meg veldig trygg**
**1**	**2**	**3**

Om evaluering av gangmønster

5 Evaluering av gangmønster

Hvordan pleier du å evaluere pasientens gangmønster før valg av egnet AFO?

	Aldri	Av og til	Som oftest	Alltid
Visuell *	○	○	○	○
Videoopptak *	○	○	○	○
Video-Vektor *	○	○	○	○
Rapport fra 3D-Ganganalyse *	○	○	○	○

6 Hvis du kunne velge, hva hadde du gjort for å evaluere pasientens gangmønster i din klinikk?

Vi ønsker å vite hvilke metoder/verktøy du synes hadde vært best egnet **i din kliniske hverdag** for å evaluere pasientens gangmønster.

7 **Om Målsettinger og funksjon**

I hvilken grad er det konkretisert en målsetting med AFOen fra henvisende lege *

- ○ Aldri
- ○ Av og til
- ○ Som oftest
- ○ Alltid

8 I de tilfeller målsetting ikke er konkretisert, pleier du selv å formulere en konkret målsetting når et barn skal få en ny AFO? *

- ◯ Aldri
- ◯ Av og til
- ◯ Som oftest
- ◯ Alltid

9 Hvor hyppig pleier du å diskutere målsetting for en AFO?

Vi ønsker å vite hvor hyppig du diskuterer målsetting med rekvirerende lege, fysioterapeut og/eller foresatte.

	diskuterer aldri målsetting	diskuterer av og til målsetting	diskuterer som oftest målsetting	diskuterer alltid målsetting
med barnet/foresatte *	◯	◯	◯	◯
med rekvirerende lege *	◯	◯	◯	◯
med fysioterapeut *	◯	◯	◯	◯

33 Vurderer du spesifikt om målsettingen med AFOen er nådd? *

- ◯ Jeg vurderer det ikke
- ◯ Jeg vurderer det av og til
- ◯ Jeg vurderer det hos de fleste av mine paseinter
- ◯ Jeg vurderer det hos alle mine pasienter

34 Hvordan evaluerer du om AFOen fungerer etter hensikten? *

Vi ønsker vite hvordan du evaluerer om AFOen har ønsket effekt på alignment og gange.

- ☐ Spør barnet/foresatte
- ☐ Visuell observasjon av gange
- ☐ Videoopptak
- ☐ Video-Vektor opptak
- ☐ 3D ganganalyse
- ☐ Annet

35 Hvordan evaluerer du om en AFO fungerer etter hensikten? *

ⓘ Dette elementet vises kun dersom alternativet «Annet» er valgt i spørsmålet «Hvordan evaluerer du om AFOen fungerer etter hensikten?»

Hvilken metode/verktøy bruker du for å evaluere om en AFO fungerer etter hensikten?

AFO for å forebygge fotfeilstillinger

Det finnes forskjellige faktorer som påvirker hvor mange timer per døgn barn skal bruker AFO.

En viktig faktor er om AFOen anses å forebygge fotfeilstillinger.

10 Vi ønsker å vite hvor hyppig leger i din klinikk spesifiserer at AFOen skal også forebygge fotfeilstillinger *

- Det blir aldri spesifisert at AFOen skal forebygge fotfeilstillinger
- Det blir av og til spesifisert at AFOen skal forebygge fotfeilstillinger
- Som oftest blir det spesifisert at AFOen skal forebygge fotfeilstillinger
- Det blir alltid spesifisert at AFOen skal forebygge fotfeilstillinger

Om alignment og konstruksjon

21 Evaluerer du legg-vinkelen i forhold til lodd-linjen for kontroll av alignment når barnet går? *

Er leggen fremoverlent eller bakoverlent i forhold til vertikalen til forskjellige tidspunkter i gangsyklus?

- Jeg evaluerer aldri legg-vinkelen
- Jeg evaluerer av og til legg-vinkelen
- Jeg evaluerer som oftest legg-vinkelen
- Jeg evaluerer alltid legg-vinkelen

22 Hvordan evaluerer du legg-vinkelen i forhold til underlaget? *

Dette elementet vises kun dersom alternativet «Jeg evaluerer alltid legg-vinkelen», «Jeg evaluerer som oftest legg-vinkelen» eller «Jeg evaluerer av og til legg-vinkelen» er valgt i spørsmålet «Evaluerer du legg-vinkelen i forhold til lodd-linjen for kontroll av alignment når barnet går?»

Tenk deg følgende situasjon:

I din klinikk treffer du et barn som ved undersøkelse av passiv bevegelighet i ankelen har **5** grader dorsalfleksjon med flektert kne og **5** grader plantarfleksjon med ekstendert kne.

23 Ville et slikt funn være en viktig indikasjon for en stiv AFO uten ankelbevegelse? *

Vi er interessert i å vite om et slikt klinisk funn spiller en viktig rolle når du velger mellom en stiv AFO og en som tillater ankelbevegelse.

- Ja, jeg synes dette er en indikasjon for bruk av stiv AFO
- Nei, jeg synes ikke at dette er indikasjon for bruk av stiv AFO

24 I et slikt tilfelle, hvordan ville du sette du opp ankelvinkelen i en stiv AFO ? *

Dette elementet vises kun dersom alternativet «Ja, jeg synes dette er en indikasjon for bruk av stiv AFO» er valgt i spørsmålet «Ville et slikt funn være en viktig indikasjon for en stiv AFO uten ankelbevegelse?»

- Jeg ville sette ankelvinkelen i AFOen i dorsalfleksjon
- Jeg ville sette ankelvinkelen i AFOen til 90 grader
- Jeg ville sette ankelvinkelen i AFOen i plantarfleksjon

25 Hvilke andre kriteria bruker du for å sikre god alignment av AFOer? *

Her følger noen spørsmål om "tuning". I denne samheng beskriver tuning utprøving av en AFO-sko kombinasjon med følgende egenskaper:

- AFOen er stiv og tillater ikke ankelbevegelse
- Ankelvinkel i AFOen skal ivareta gastrocnemius lengde og settes i plantarfleksion ved behov.
- Korrekt legg-vinkel i forhold til underlaget er oppnått ved å bygge om skosålen.

26 Er du kjent med denne metoden for "tuning" AFO? *

- ○ Ja
- ○ Nei

27 Har du tilpasset stive AFOer med ankelen i plantarfleksion som ble "tuned" ved å endre på skosålen? *

Dette elementet vises kun dersom alternativet «Ja» er valgt i spørsmålet «Er du kjent med denne metoden for "tuning" AFO?»

- ○ Ja
- ○ Nei

28 Når du har gående barn med CP som har stram gastrocnemius, hvor hyppig tilpasser du slike "tuned" AFOer? *

Dette elementet vises kun dersom alternativet «Ja» er valgt i spørsmålet «Har du tilpasset stive AFOer med ankelen i plantarfleksion som ble "tuned" ved å endre på skosålen?»

- ○ Sjelden
- ○ Av og til
- ○ Som oftest
- ○ Alltid

29 Hvordan finner du korrekt alignment når du "tuner"? *

Dette elementet vises kun dersom alternativet «Ja» er valgt i spørsmålet «Har du tilpasset stive AFOer med ankelen i plantarfleksion som ble "tuned" ved å endre på skosålen?»

- ☐ Visuell observasjon
- ☐ Video-opptak
- ☐ Video Vektor opptak
- ☐ 3D ganganalyse

30 Dersom du velger å ikke tilpasse AFOer med tuning, hva er grunnen? *

Dette elementet vises kun dersom alternativet «Ja» er valgt i spørsmålet «Er du kjent med denne metoden for "tuning" AFO?»

Dette gjelder barn som har stram gastrocnemius.

- ☐ Jeg foretrekker AFOer med ledd også i slike tilfeller
- ☐ "Tuning" fungerer i teorien, men i praksis kan vi ikke bygge om alle skoene til pasienten
- ☐ Lav aksept hos pasienter for en slik AFO-sko kombinasjon
- ☐ Utprøving tar for lang tid
- ☐ Jeg mangler opptaksutstyr
- ☐ Jeg mangler kompetanse
- ☐ Annet

31 Andre grunner for å velge bort stiv AFO-sko kombinasjon med "tuning" *

Dette elementet vises kun dersom alternativet «Annet» er valgt i spørsmålet «Dersom du velger å ikke tilpasse AFOer med tuning, hva er grunnen?»

Hvilken AFO til hvilket gangmønster?

Nå følger en seksjon i spørreskjemaet der vi ønsker å vite hvilken type AFO du ville anbefale til barn med forskjellige gangmønstre. Vi vet at det er mange faktorer som som påvirker valget, og et slikt mønster i seg selv har ingen fasit. Det er likevel fint om du kan prøve å bidra med dine tanker rundt bruk av AFO i forskjellige situasjoner.

Gangmønster i barn med Cerebral parese (Papagergiou,2019)

Type 1	*Type 2*	*Type 3*	*Type 4*	*Type 5*	*Type 6*
Droppfot	***Spissfot******	***Genu recurvatum***	***Jump kne***	**Tilsynelatende spissfot**	***Crouch***
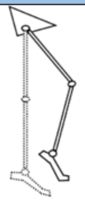	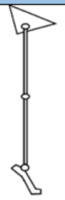	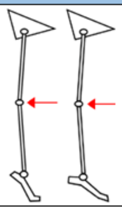	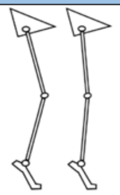	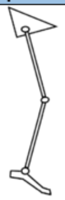	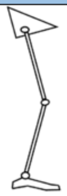
Droppfot i sving men adekvat ankel ROM i standfasen.	Ankel i spissfot i standfasen. Full ekstensjon i kne og hofte.	Full ekstensjon i kne eller overstrekk. Nedsatt motorisk kontroll i ankel som fører til plantarfleksjon eller redusert dorsalfleksjon og bremser framåt bevegelsen av tibia.	Ankel i spissfot. Fotisett med flektert hofte og kne, fulgt av tidlig ekstensjonsbevegelse i hofte og kne.	Ankel med normal ROM, forøket fleksjon i hofte og kne i standfasen.	Fleksjonsmønster i hofte og kne. Forøket dorsalfleksjon i støtfasen

*ROM = bevegelsesomfang
**Spissfot = begrenset ankel ROM grunnet kontraktur og/eller spastisitet

Papageorgiou E et.al *Systematic review on gait classifications in children with cerebral palsy*: An update. Gait Posture. 2019;69:209-23.

Marker hvilken AFO-egenskap som du vurderer egnet til hvert gangmønster

Nedover får du en liste med forskjellige AFO-typer og egenskaper.

Bortover kan du velge gangmønstre som hver type/egenskap kan være egnet for.

		Type 1, droppfot	Type 2, spissfot	Type 3, Genu Recurvatum	Type 4, jump kne	Type 5, til-synelatende spissfot	Type 6, Crouch	Brukes ikke
	Lycra *	☐	☐	☐	☐	☐	☐	☐
11	FES (elektrisk stimulering) *	☐	☐	☐	☐	☐	☐	☐
12	Uten ledd, dynamisk/fjærende, pre-fabrikkert (t.eks.ToeOff©) *	☐	☐	☐	☐	☐	☐	☐
13	Uten ledd, dynamisk/fjærende, indi-viduelt fremstilt (t.eks.Springer©) *	☐	☐	☐	☐	☐	☐	☐
14	Leddet, åpen dorsalfleksjon, plantarfleksjonsstopp *	☐	☐	☐	☐	☐	☐	☐
15	Leddet, fjærbelastet ledd (t.eks.NeuroSwing©, TrippleAction©) *	☐	☐	☐	☐	☐	☐	☐
16	Uten ledd, ingen ankelbevegelse *	☐	☐	☐	☐	☐	☐	☐
	Fleksibel fotplate *	☐	☐	☐	☐	☐	☐	☐
	Stiv fotplate *	☐	☐	☐	☐	☐	☐	☐
	Ombygging av skoen for "tuning" *	☐	☐	☐	☐	☐	☐	☐

32 Med hvem pleier du å diskutere konstruksjonen av AFO?

Hensikten er å finne ut hvem som bidrar i utvalgsprosessen om AFOens mekaniske egenskaper. Er dette er et valg du hovedsakelig treffer alene, eller pleier dette å være en prosess som involverer andre?

Her beskriver "konstruksjon" de mekaniske egenskaper som for eksempel bruk av ledd, om AFOen er prefabrikkert eller individuelt framstilt, eller hvordan den skal bli produsert. Gjelder ikke kosmetiske valg som mønsteret på temoplast.

	AFO kon-struksjonen diskuterer jeg aldri	AFO kon-struksjonen diskuterer jeg av og til	AFO kon-struksjonen diskuterer jeg som oftest	AFO kon-struksjonen diskuterer jeg alltid
Med barnet/foresatte *	○	○	○	○
Med rekvirerende lege *	○	○	○	○
Med fysioterapeut *	○	○	○	○
Med ingeniør-kolleger *	○	○	○	○
Med teknikker-kolleger *	○	○	○	○

17 Kommentarer til valg av AFOer til gående barn med CP *

Hvis du har andre kommentarer til valg av AFOer til forskjellige gangmønstre, kan du skrive det her:

Om bruk av Lycra

Hvordan vil du beskrive din kompetanse ved bruk av Lycra ortoser, uavhengig av pasientdiagnosen? *

- ○ Lav, jeg har ikke mye praksis med bruk av Lycra ortoser
- ○ God, jeg bruker Lycra ortoser når det er aktuelt

Bruker du Lycra ortoser som alternativer til AFOer for gående barn med CP? *

Dette elementet vises kun dersom alternativet «God, jeg bruker Lycra ortoser når det er aktuelt» er valgt i spørsmålet «Hvordan vil du beskrive din kompetanse ved bruk av Lycra ortoser, uavhengig av pasientdiagnosen?»

- ○ Ja, jeg bruker Lycra ortoser for gående barn med CP
- ○ Nei, jeg bruker ikke Lycra ortoser for gående barn med CP

Hva er hensikten med å bruke Lycra ortoser til gående barn med CP? *

Dette elementet vises kun dersom alternativet «Ja, jeg bruker Lycra ortoser for gående barn med CP» er valgt i spørsmålet «Bruker du Lycra ortoser som alternativer til AFOer for gående barn med CP?»

Bruker du Lycra ortoser når det foreligger kontraktur i leggmuskulaturen? *

Dette elementet vises kun dersom alternativet «Ja, jeg bruker Lycra ortoser for gående barn med CP» er valgt i spørsmålet «Bruker du Lycra ortoser som alternativer til AFOer for gående barn med CP?»

- ◯ Ja, Lycra ortoser kan fungere godt når det foreligger kontraktur
- ◯ Nei, når det foreligger kontraktur i leggmuskulaturen bruker jeg ikke Lycra ortoser.

Bruker du Lycra ortoser når det foreligger spastisitet i leggmuskulaturen? *

Dette elementet vises kun dersom alternativet «Ja, jeg bruker Lycra ortoser for gående barn med CP» er valgt i spørsmålet «Bruker du Lycra ortoser som alternativer til AFOer for gående barn med CP?»

- ◯ Ja, Lycra ortoser kan fungere godt når det foreligger spastisitet
- ◯ Nei, når det foreligger spastisitet i leggmuskulaturen bruker jeg ikke Lycra ortoser.

Om bruk av FES, funksjonell elektrisk stimulering

Hvordan vil du beskrive din kompetanse ved bruk av funksjonelle elektriske stimulerende ortoser (FES), uavhengig av pasientdiagnosen? *

- ◯ Lav, jeg har ikke mye praksis FES ortoser
- ◯ God, jeg bruker FES ortoser når det er aktuelt

Bruker du FES-ortoser til gående barn med CP? *

Dette elementet vises kun dersom alternativet «God, jeg bruker FES ortoser når det er aktuelt» er valgt i spørsmålet «Hvordan vil du beskrive din kompetanse ved bruk av funksjonelle elektriske stimulerende ortoser (FES), uavhengig av pasientdiagnosen?»

- ◯ Ja, jeg bruker FES ortoser til gående barn med CP
- ◯ Nei, jeg bruker ikke FES ortoser til gående barn med CP

Hva er hensikten med å bruke FES ortoser til gående barn med CP? *

Dette elementet vises kun dersom alternativet «Ja, jeg bruker FES ortoser til gående barn med CP» er valgt i spørsmålet «Bruker du FES-ortoser til gående barn med CP?»

Bruker du FES ortoser når det foreligger kontraktur i leggmuskulaturen? *

Dette elementet vises kun dersom alternativet «Ja, jeg bruker FES ortoser til gående barn med CP» er valgt i spørsmålet «Bruker du FES-ortoser til gående barn med CP?»

- ◯ Ja, FES ortoser kan fungere godt til barn som har kontraktur
- ◯ Nei, jeg bruker ikke FES ortoser til barn som har kontraktur

Bruker du FES ortoser når det foreligger spastisitet i leggmuskulaturen? *

Dette elementet vises kun dersom alternativet «Ja, jeg bruker FES ortoser til gående barn med CP» er valgt i spørsmålet «Bruker du FES-ortoser til gående barn med CP?»

- ○ Ja, FES ortoser kan fungere godt til barn som har spastisitet
- ○ Nei, jeg bruker ikke FES ortoser til barn som har spastisitet

Hvordan påvirker ålder og aktivitet AFO egenskaper?

Hvilke AFO egenskaper mener du er viktig for en 5-åring som går i barnehage? *

Hvilke AFO egenskaper mener du er viktig for en 10-åring som går på skolen? *

Hvilke AFO egenskaper mener du er viktig for en ungdom? *

Hvilke AFO egenskaper mener du er viktig for et barn/ungdom som er fysisk aktiv? *

Her betyr "fysisk aktiv" noe aktivitet utover det hverdagslige, som å spille fotball, gå på ski eller annet.

Hvor hyppig ser du behov for AFOer som er fremstilt spesifikt for fysisk aktivitet? *

- AFO til bruk i aktivitet som har andre egenskaper enn den som brukes til vanlig.
- Dette gjelder kun barn som driver med aktiviteter som fotball eller lignende.

- ○ Jeg ser aldri behov for en aktivitets-AFO
- ○ Jeg ser av og til behov for en aktivitets-AFO
- ○ Jeg ser som oftest behov for en aktivitets-AFO
- ○ Jeg ser alltid behov for en aktivitets-AFO

Kommentarer

Om det finnes andre faktorer som bestemmer utvalgsprosessen av AFOer, og evalueringsprosessen av utleverte AFOer, setter vi pris på ditt innspill.
